# Sow-Piglet Nose Contacts in Free-Farrowing Pens

**DOI:** 10.3390/ani9080513

**Published:** 2019-07-31

**Authors:** Katrin Portele, Katharina Scheck, Susanne Siegmann, Romana Feitsch, Kristina Maschat, Jean-Loup Rault, Irene Camerlink

**Affiliations:** Institute of Animal Welfare Science, University of Veterinary Medicine, Vienna, Veterinaerplatz 1, 1210 Vienna, Austria

**Keywords:** sow, piglet, behaviour, mother–offspring, nosing, free-farrowing, positive welfare, contact, maternal care, recognition

## Abstract

**Simple Summary:**

The mother–offspring interaction is important for the young’s development, yet it is rarely taken into account in farm animals for whom restricted contact and early separation from the mother are common. Nose-to-nose contact is a poorly understood form of social interaction between pigs. We investigated the occurrence and type of nose-to-nose contacts and whether and why it could differ between sows and piglets. Twenty-two sows and their 249 piglets were observed in free-farrowing pens for the first three weeks of the piglets’ life. Sows and their piglets made nose contact with each other every 10 min, on average. Heavier piglets made more nose contact with the sow than lighter piglets in their first week of life. Unexperienced mothers nosed their piglets more in the second week of the piglets’ life. Allowing sows and piglets to freely make nose contact may improve mother–young relationships and piglets’ development, possibly benefiting animal welfare and productivity.

**Abstract:**

Nose contact is a frequent form of social behaviour in pigs, but the motivational reasons underlying this behaviour remain unclear. We investigated the frequency, direction and type of sow–piglet nosing behaviour and its association with sow and piglet traits. Social nosing behaviour was recorded by live observations and video recordings in 22 sows and their 249 piglets in free-farrowing pens once weekly during the first three weeks after farrowing (3 times 30 min of observations per litter). Piglet-to-sow nosing occurred on average 32.8 ± 2.35 times per 30 min per litter. Heavier piglets at one week of age nosed the sow more than lighter piglets (*p* = 0.01). Piglet-to-sow nosing was unrelated to the piglet’s sex or teat order. Sow-to-piglet nosing occurred on average 3.6 ± 0.53 times per 30 min, and this was unrelated to litter size. Primiparous sows nosed their piglets more in the second week after farrowing. Litters in which piglet-to-sow nosing occurred more showed less variation in the expression of this behaviour across the weeks. Social nosing between sow and piglets deserves further research to understand the positive implications of this behaviour for sow and piglet welfare.

## 1. Introduction

Mother–offspring contact has crucial effects on offspring development. Studies in rodents have shown that grooming and licking of the pups by the mother have a major impact on social and neurological development and the stress-coping abilities of the offspring [1,2,3]. In farm animal husbandry, mother–offspring contact is often greatly restricted or even absent. Separation from the young enables an increase in the commercial output of the dam, whether this is the quantity of milk for human consumption or the number of offspring weaned per year. As a consequence, farm animals are often separated from the mother far earlier than common in nature. Moreover, the use of constraining housing systems, such as farrowing crates for sows, restrict the dam’s movements and thereby her ability to initiate interactions with her offspring.

Possibly as a consequence, the mother–offspring relationship has received only little research attention in farm animals (calves [4]; sheep [5]; pigs [6]), with the main focus on the negative effects of separation from the dam [7]. This has left a knowledge gap regarding naturally occurring mother–offspring interactions and the importance of allowing the expression of maternal care in farm animals.

In wild boar, sow–piglet interactions are mainly in the form of suckling and nose contact [8]. Nose contact in pigs has been suggested to be essential in facilitating mother–offspring bonding [8,9,10]. Social nosing may also aid in communicating needs, e.g.**,** nutritional needs, as suggested by the positive association between nosing and milk intake [11]. The frequency of piglet-to-sow nosing has been shown to increase during the first five days of life and then progressively decline over the first four weeks of life [10,12]. This decline coincides with the time piglets in nature would start to follow their mother outside the nest [13].

Mothers can also adjust their care to their offsprings’ needs. For example, ewes are more attentive when their lambs experience pain [14]. However, in pig husbandry, the majority of sows are currently housed in farrowing crates [15], which restrict the sow’s movement to standing, sitting and lying without being able to turn around or freely interact with their piglets. Sows in farrowing crates have indeed fewer interactions with their piglets than sows that can move freely [16,17,18]. The inability of sows to properly interact with the piglets might also be, in part, a reason for savaging of piglets by the sow [19], a serious welfare concern that is mostly seen in primiparous sows, i.e.**,** gilts [20]. A greater understanding of the role of maternal care and mother–offspring communication is, therefore, important to animal welfare.

The aim of the current study was to examine sow–piglet nosing behaviour in free-farrowing pens and its associations to sow and piglet traits such as litter size, sow parity, and piglets’ weight, teat order and sex.

## 2. Materials and Methods

All methods and animal use within this study were approved by the institutional ethics committee of the University of Veterinary Medicine, Vienna (protocol number 05/09/2018) in accordance with Good Scientific Practice guidelines and national legislation.

### 2.1. Animals and Housing

Observations took place at the pig research and teaching farm of the University of Veterinary Medicine, Vienna, Austria. Twenty-two sows (Large-White × Landrace) and their litters (249 piglets) were observed over two farrowing batches five weeks apart. Batch 1 consisted of 14 sows and 160 piglets while Batch 2 consisted of eight sows and 89 piglets. Sows were mainly in their first parity (45%) or second parity (18%), and the remaining sows were in their third (9%), fifth (14%), sixth (9%) or seventh (5%) parity. Batch 1 and 2 had five primiparous sows each. Sows were moved from their group housing into one room (same room for both batches) and housed in BeFree farrowing pens (Schauer Agrotronic GmbH, Prambachkirchen, Austria) one week before expected parturition. Sows had previously also farrowed in free-farrowing pens. The BeFree pen had a floor space of 2.22 × 2.86 m in total (6.35 m^2^, with 4.2 m^2^ for the sow) with plastic slatted floor and a concrete lying area. Sows had a feeder with drinker and a hay rack and a bar on one side of the wall to facilitate lying down. They had ad libitum access to water and were fed dry commercial sow feed (pellets) at 7:00, 11:30 and 15:30 and received hay daily in a rack. Sows were not crated before, during or after farrowing, except when required for short-term handling of the sow or piglets. The piglets had a creep area of 1.25 × 0.61 m (0.76 m^2^), one drinker (ad libitum water) and received a commercial piglet feed (pre-starter meal) from seven days of age. Lights were on between 07:00 and 16:00 and the temperature was set at 20 °C. Average litter size was 11.3 ± 0.2 (SE) piglets (range 8–13). Cross-fostering was applied if the number of piglets exceeded the number of functional teats of the sow. In the first week of life, piglets’ teeth were grinded to reduce facial injuries in the piglets, but tails were kept intact. Males were castrated at 10–14 days post-partum under general anaesthesia. The piglets were ear-tagged at approximately 19 days of age.

### 2.2. Data Collection

Animals were observed at the end of weeks 1, 2 and 3 of lactation. Sows farrowed at most four days apart from each other, and sows were on average (means ± SE), 8.3 ± 2.1 days, 13.4 ± 1.3 days and 21.0 ± 1.5 days in lactation. In Batch 1, 12 sows were observed by live observations and two sows from the same room were observed by video recordings due to the lack of an observer owing to health reasons at those dates. To assess the potential effect of the observation method, eight sows from a previous batch (here referred to as Batch 2) were observed from video recordings. As these sows had already been weaned, it was not possible to mark, sex or weigh the piglets. Differences between the batches are shown in Table 1.

**Figure 1:**
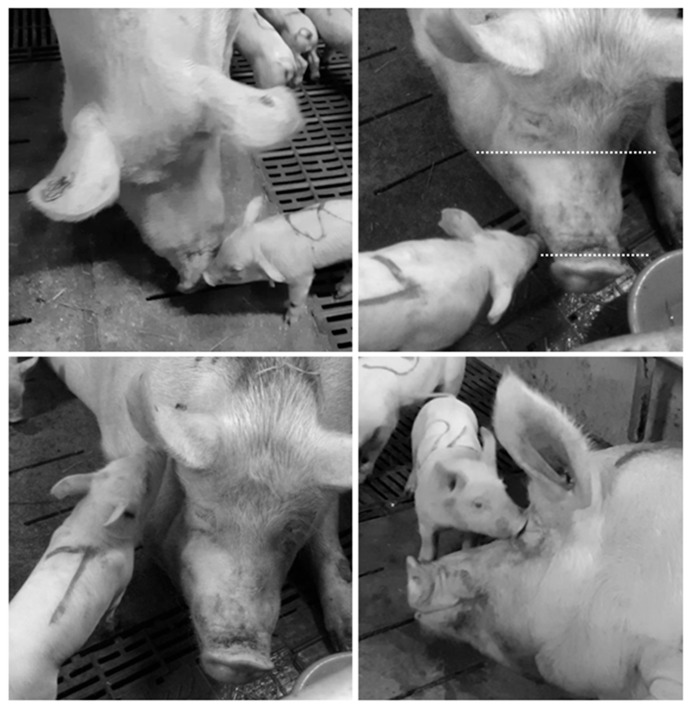
Target areas of the four types of piglet-to-sow nosing behaviours: nose-to-nose (top left), nosing snout, with snout area indicated between dotted lines (top right), nosing ear (bottom left), and nosing head (bottom right).

We recorded the frequency of four piglet behaviours directed toward the sow (nose-to-nose, nosing snout, nosing ear, and nosing head, Figure 1) and one sow behaviour directed toward the piglets (sow-to-piglet nosing) (Table 2). Nosing was counted if physical contact occurred. Multiple contacts performed by the same individual <5 sec apart were counted as one occurrence. We also recorded whether the nosing behaviour occurred during a suckling bout or within one minute after a suckling bout, for Batch 1 only (Table 2). Each litter was continuously observed for three blocks of 10 min each (total 30 min) in weeks 1, 2 and 3 of lactation, resulting in a total of 90 min of behavioural observations per litter. Sampling only took place when more than 50% of the piglets in a litter were active and the sow was not feeding at the beginning of observation. Otherwise, the litter was observed at the next observation time that day when the litter was active and the sow was not feeding. This resulted in 2089 occurrences of piglet-to-sow behaviour and 236 occurrences of sow-to-piglet behaviour (Table 1).

Behaviours during live and video observations were recorded using the ‘Ad libitum’ sampling option in the app Animal Behaviour Pro version 1.2 (University of Kent, Canterbury, UK), installed on iPad or iPhone (iOS devices).

Live behavioural observations were carried out between 13:00 to 16:00 h. Piglets were marked with a marker pen for individual identification in the morning before observations, leaving at least a 30 min break before the start of the live and video observations. As piglets had no ear tag, the identification with the back number applied only to that observation day (i.e., piglets could not be re-identified across observation weeks).

Videos were recorded using overhead cameras (GV-BX 1300-KV, Geovision, Taipei, Taiwan) in a waterproof case (HEB32K1, Videotec, Schio, Italy) placed with a top-down view ~5 m above the pen. The images were recorded with 1280 × 720 pixel resolution at 30 frames per second. Recordings at the end of weeks 1, 2 and 3 of lactation, between 13:00 to 16:00 h, were selected and watched using Windows Media Player. Video observations were conducted using the same protocol as for live observations. However, piglets of Batch 2 were not marked and thus not individually identifiable. Therefore, data from Batch 2 consist of frequencies of behaviour for the litter rather than individual level. Behaviour recorded from video recordings can sometimes differ from behaviour recorded through live observations due to human presence during live observations. The sows at the research unit are selected for docile behaviour towards humans as students work with them frequently while the sows are unrestrained in a free-farrowing pen. Comparisons of the behaviour recorded through videos compared to live observations showed that there was no significant difference between the two recording methods in the frequency of sow-to-piglet contact (*t*-test: t_64_ = 0.64; *p* = 0.52) and piglet-to-sow contact (*t*-test: t_64_ = 1.59; *p* = 0.12).

The live observations were conducted by three observers simultaneously while each rotated between four litters, whereas video observations were conducted by a single observer. Inter-observer reliability was calculated for all four observers from a 10-min live observation block with the package “irr” [21] in R version 3.5.1 [22] using a two-way mixed, agreement, single-measures Intraclass Correlation Coefficient (ICC). The results showed excellent agreement for overall nosing (98.6%), as well as for the five behaviours separately (nose-to-nose: 89.6%, nosing snout: 93.4%, nosing ear: 94.1%, nosing head: 100%, sow-to-piglet nosing: 100%).

Piglets of Batch 1 (*n* = 160) were sexed and weighed in weeks 1, 2 and 3 of lactation. There were 84 males (52%) and 76 females (48%). Piglet teat order was recorded opportunistically across the observation days in Batch 1, resulting in two to three occurrences per piglet in total. As weight would be similar for piglets across the weeks, while piglet identity was unknown, only the weight at week 1 was analysed (when the piglet was the experimental unit) to avoid pseudoreplication. Average litter weight (by week) was included in the analysis of sow-to-piglet behaviour.

The data have been made available online (see Supplementary Materials statement).

### 2.3. Data Analysis

The coefficient of variation (CV) was calculated for the total amount of piglet-to-sow nosing of the litter and for sow-to-piglet nosing across the lactation weeks.

Statistical analyses were performed using SAS version 9.4 (Statistical Analysis Software, SAS Institute, USA). The experimental unit for the frequency of piglet-to-sow nosing and sow-to-piglet nosing was the litter.

Differences in piglet-to-sow nosing between the lactation weeks (i.e., observation weeks) were analysed at litter level using a linear mixed model (MIXED Procedure) with piglet-to-sow nosing behaviour as a response variable and, as fixed variables, the litter size and lactation week. The lactation week was included as a repeated variable with the litter nested within the batch specified as a subject to account for the repeated observations across the lactation weeks per litter.

Frequency of sow-to-piglet nosing was analysed using a linear mixed model (MIXED Procedure) with the fixed effects of sow parity (primiparous or multiparous), week of lactation (1–3), litter size, average litter weight and the interaction sow parity × week of lactation. The lactation week was included as a repeated variable with the sow nested within the batch specified as subject to account for the repeated observations across the lactation weeks per sow.

The relationships between piglet-to-sow nosing (nose-to-nose, nosing snout, nosing ear, nosing head, and total frequency) and the piglets’ body weight, sex and teat order were analysed with the piglet as the experimental unit (data of Batch 1 only, *n* = 160) using linear mixed models. The fixed effects were sex (male or female), body weight week 1, average teat order, and the interaction of sex × body weight week 1. The litter was included as a random effect to account for the lack of independence between the piglets in the same litter.

Variables were omitted from the models if they had a *p*-value >0.10 and only if their removal improved the model fit as assessed through the Akaike information criterion (AIC) and Bayesian Information Criterion (BIC). For each model, residuals of the response variables were assessed for the normality of their distribution (Shapiro–Wilk test statistics), homogeneity and outliers. Across all variables, ten data points with frequencies greater than three standard deviations from the mean were identified as outliers. They were checked against the original data and were determined as unlikely to be recording errors and, therefore, were retained. Pearson correlations were conducted in order to assess relationships between the average frequency of piglet-to-sow nosing and sow-to-piglet nosing, and between CV and average frequency of nosing. Data are presented as Least Square means (LS-means) with standard errors (SE). *p*-values <0.05 were considered statistically significant, whereas *p*-values between 0.05 and 0.10 are stated with their actual values as tendencies.

## 3. Results

### 3.1. Piglet-To-Sow Nosing Behaviour

**Figure 2:**
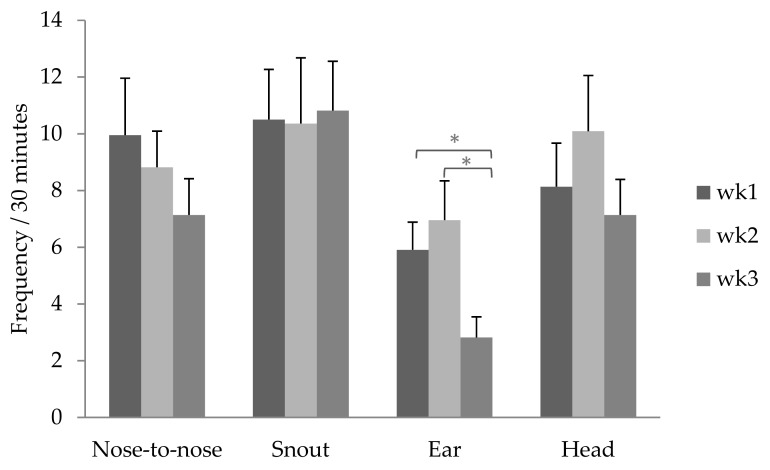
Average frequency of the four types of piglet-to-sow nosing behaviour (*n* = 22 litters) by observation week (30 min per week). Values are LS-means with SE. * significant difference indicated by *p* < 0.05.

The frequency of piglet-to-sow nosing was, on average, 32.8 ± 2.35 occurrences per 30 min per litter, or the equivalent of 2.9 ± 0.21 times per 30 min per piglet. Piglet-to-sow nosing averaged 34.0 ± 4.05 occurrences in week one, 35.8 ± 4.05 occurrences in week two, and 27.5 ± 4.05 occurrences in week three, with no significant differences between weeks (F_2,42_ = 1.18; *p* = 0.32). For all weeks, the frequency with which individual piglets directed nosing to the sow was not reciprocated by the sow towards the individual piglets in the same frequency (correlation between nosing given and nosing received: *r* = 0.03; *p* = 0.56). Nosing the ear occurred least in week three (F_2,42_ = 4.20; *p* = 0.02), whereas nose-to-nose contact and nosing of the snout and head did not significantly differ across the lactation weeks (Figure 2). Across the three weeks, 12.45% of piglet-to-sow nosing occurred during a suckling bout, 8.15% within one minute after a suckling bout and 79.40% between suckling bouts, with no statistically significant differences between weeks.

**Figure 3:**
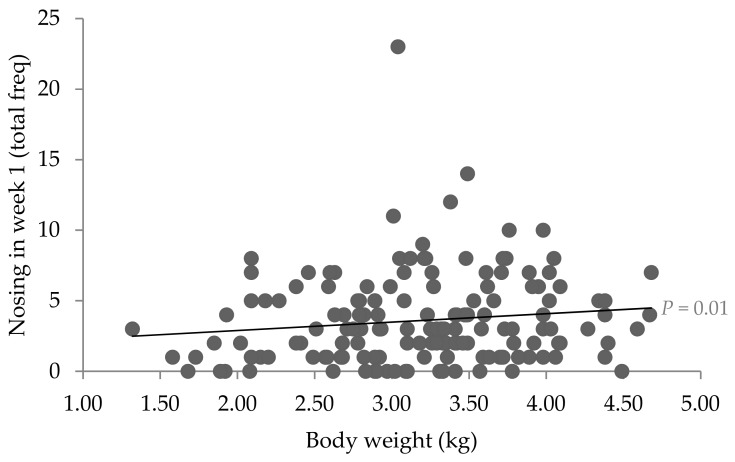
Relationship between piglet body weight and the frequency of piglet-to-sow nosing behaviour in the first week of life (*n* = 160 piglets).

In the first week of life, piglets weighed on average 3.18 ± 0.054 kg (means ± SE; range 1.32–4.68 kg), whereby heavier piglets performed more nosing toward the sow (b = 0.78 ± 0.641; F_1,111_ = 6.18; *p* = 0.01; Figure 3). The frequency of piglet-to-sow nosing did not differ between males (4.07 ± 0.566 occurrences) and females (3.44 ± 0.573 occurrences) (F_1,111_ = 1.23; *p* = 0.27) or according to the interaction of sex × body weight wk 1 (F_1,111_ = 0.79; *p* = 0.38). Piglet-to-sow nosing was not related to the piglet’s teat order (F_7,111_ = 0.76; *p* = 0.62). Moreover, there was no significant relationship between piglet weight and their average teat order (*r* = −0.02; *p* = 0.67).

### 3.2. Sow-To-Piglet Nosing Behaviour

**Figure 4:**
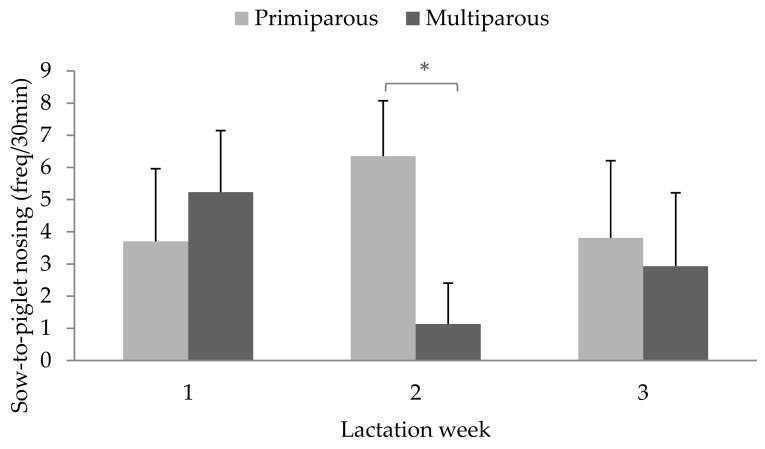
Frequency of sow-to-piglet nosing behaviour (per 30 min) for primiparous sows (i.e., gilts; *n* = 10) and multiparous sows (*n* = 12) throughout lactation. Values are LS-means with SE. * *p* < 0.05.

Sows nosed their piglets on average 3.6 ± 0.53 times (range: 0–24) per 30 min. The frequency with which the sow nosed her litter did not correlate with the frequency of nosing that the piglets in the litter directed towards the sow (*r* = −0.19; *p* = 0.40). Sow-to-piglet nosing did not differ according to the number of piglets in the litter (F_1,11_ = 0.48; *p* = 0.50), the average litter weight (F_1,23_ = 0.16; *p* = 0.69) or the lactation week (week 1: 4.04 ± 0.91 occurrences, week 2: 3.30 ± 0.91 occurrences, week 3: 3.59 ± 0.91 occurrences; F_2,23_ = 0.08; *p* = 0.92). The frequency of sow-to-piglet nosing did not differ according to sow parity (primiparous: 4.62 ± 1.03; multiparous: 3.10 ± 0.73; F_1,11_ = 1.26; *p* = 0.29), but tended to differ according to the interaction of parity and lactation week (F_2,23_ = 2.90; *p* = 0.07; Figure 4), with primiparous sows showing significantly more sow-to-piglet nosing in the second week of lactation compared to multiparous sows (*p* = 0.02), and a trend for primiparous sows to perform more sow-to-piglet nosing in week two compared to week one (*p* = 0.06).

### 3.3. Variation in Social Nosing Behaviour Across the Lactation Period

**Figure 5:**
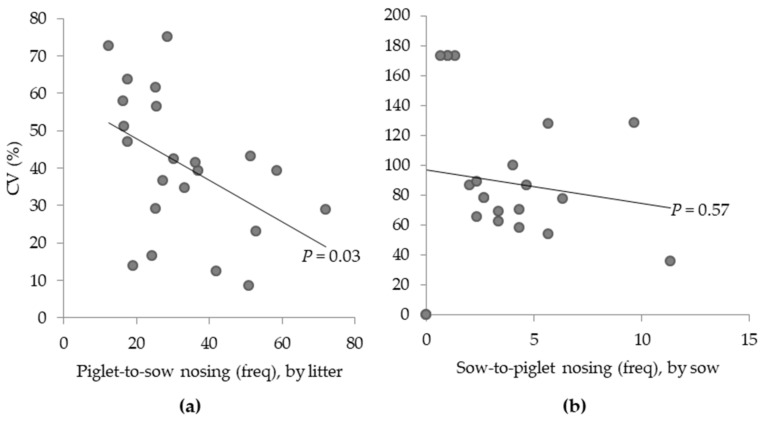
Coefficient of variation (CV; %) for the average frequency (per 30 min of observation) of piglet-to-sow nosing (**a**) and sow-to-piglet nosing (**b**) behaviours across the lactation period (*n* = 22 litters).

The variation in piglet-to-sow nosing within the litter across the lactation weeks, as determined by the coefficient of variation (CV), was, on average, 40.7% ± 4.06% (Figure 5a), and ranged from 8.5% to 75.2% between litters. The CV of piglet-to-sow nosing and its average frequency were significantly negatively correlated across the lactation period (*r* = −0.46; *p* = 0.03; Figure 5a). The CV for sow-to-piglet nosing across the lactation weeks was on average 89.1% ± 10.89% (Figure 5b), and ranged from 0% to 173.2% between sows; two sows did not nose their piglets at all during observations (frequency and CV of 0; these were a primiparous and a multiparous sow from video and live observations, respectively). One primiparous sow in the live observations (most right data point) consistently nosed her piglets frequently, on average, once every 2.5 min. The CV for sow-to-piglet nosing was not correlated with its average frequency (*r* = −0.13; *p* = 0.57; Figure 5b).

## 4. Discussion

Social nosing was frequently observed between the sow and her piglets, and for piglet-to-sow nosing behaviour each of the four subtypes were observed. Heavier piglets nosed the sow more than lighter piglets in the first week of life. Primiparous sows showed more nosing behaviour toward their piglets in the second week of lactation. These findings altogether suggest a role for social nosing between the sow and her piglets in social recognition, communication and maternal care.

### 4.1. Piglet-To-Sow Nosing Behaviour

Piglets frequently initiated nose contact with the sow. On average a piglet made a nose contact with the sow 2.9 times in the 30 min of observations. This is slightly higher than in the study of Stangel and Jensen [10], who recorded between one and two piglet-to-sow nose contacts per 2 h in piglets up to 10 days of age housed in a large outdoor enclosure (7–13 ha), and Blackshaw and Hagelsø [23] who recorded around 2.4 piglet-to-sow nose contacts per hour in free-farrowing pens (2.6 × 2.3 m). The higher occurrence of piglet-to-sow nosing in the current study may be due to different factors such as, amongst others, a larger litter size as compared to the aforementioned studies or the inclusion of nosing the ear and head rather than solely nose–nose contacts, as was the case in previous studies. Indeed, the four subtypes of piglet-to-sow nosing behaviours that we recorded were observed to be similarly prevalent, and constant over the first three weeks of life apart from piglets nosing the sow’s ear, which reduced in week three. A reduction in contact was expected as piglets may divert to other activities as they get more active, and the finding that snout contacts remain stable across the weeks may be related to its wider function in communication [8].

We initially predicted that lighter-weight piglets, who may suffer from lack of milk, would initiate more social nosing with the sow to give an honest signal of their need, as they do with vocalizations [24,25]. In contrast, we found that in the first week of life, heavier piglets performed more nosing toward the sow. Heavier piglets may be healthier and stronger and have more energy left for activity, including for interacting with the sow. The causality of the relationship between body weight and nosing is, however, not necessarily unidirectional as previous work found that weaned pigs that received more nosing from others had a better growth performance [26], although in the present study, piglet-to-sow nosing did not appear to be necessarily reciprocated by the sow. Piglet-to-sow nosing has also been related to suckling and milk ejection [11,16,23]. For example, Blackshaw and Hagelsø [23] reported that 36% of the piglet-to-sow nosing was associated with lactation. Nevertheless, we did not find a difference in the frequency of nosing during or within one minute after a suckling bout in the current study.

### 4.2. Sow-To-Piglet Nosing Behaviour

Sows nosed their piglets on average 3.6 times per 30 min. This is again higher than reported by Stangel and Jensen [10], which may be due to the fact that in our study, nose contact toward any body part of the piglet was counted, not only nose-to-nose contact. Earlier studies in semi-natural enclosures and free-farrowing pens reported a decrease in sow-to-piglet nosing in the first ten days post-partum [10,23], and between the first and fourth week of lactation [12].

Primiparous sows delivered more nosing toward their piglets in the second week after farrowing as compared to multiparous sows, and this also tended to be higher than during their first week after farrowing. Primiparous sows can be more restless [27,28] and insecure during and after their first farrowing [29], which can increase sow-to-piglet nosing behaviour [19]. Restlessness is also associated with savaging behaviour in primiparous sows [28], which is a considerable welfare concern as it may lead to substantial piglet mortality (up to 25% mortality [19]). However, Ocepek and Andersen [30] found that sows who communicated more with their piglets, including sniffing, grunting and nudging, had a lower mortality rate in their litter, partly due to less piglet crushing. It might be that the investigation and recognition of the young [31], or other forms of maternal behaviour that sow-to-piglet nosing underlies, takes some time to establish during their first maternal experience [32]. When identification is established, the need of the sow to nose her piglets might be less. These results suggest that sow-to-piglet nosing behaviour is a form of maternal care, possibly facilitating the formation of the mother–offspring bond, which sows learn from experience with their first litter and they retain and retrieve this skill more quickly in subsequent farrowing episodes.

The amount of nosing behaviour that the piglets directed towards the sow was unrelated to the amount of nosing the sow directed to her piglets, and did not show reciprocity between individual piglets and the sow within a given week. Sow-to-piglet nosing did, however, occur infrequently and more observation hours per sow would be needed to draw conclusions upon the reciprocity of nosing behaviour between the sow and her piglets. Similarly, more data on sow-to-piglet nosing would be needed to meaningfully analyse whether the sow directs more nosing towards the weaker piglets in the litter. Based on the current frequency of sow-to-piglet nosing in relation to the litter size, at least 3 h of behavioural observations per sow would be required to assess these two above-mentioned questions.

### 4.3. Variation in Nosing Behaviour

Considerable within- and between-litter variation was seen in the frequency of social nosing, based on the calculated CVs and a relatively large range. Litters that displayed a greater frequency of piglet-to-sow nosing showed less variation across the weeks, suggesting that piglet-to-sow behaviour is consistent during lactation at litter level. Sow-to-piglet nosing behaviour would benefit from more observation time per sow, as this behaviour occurred infrequently, and it was not observed at all for 2 out of the 22 sows, which is a similar result to those reported from Blackshaw and Hagelsø [23]. The naturally occurring variation in sow-to-piglet behaviour, which is related to mothering abilities [30], could be used to breed good mothers for instance.

## 5. Conclusions

Sow–piglet nose contact is a commonly occurring behaviour when sows and piglets are able to freely interact. As pointed out in several studies, sow–piglet nose contacts may be of considerable importance to the development of piglets and to allow for the expression of maternal care by the sow. We would like to emphasize the importance of this “*most perplexing behaviour*” [16] and the need to study social nosing in pigs in its own right as a key social communication behaviour. With the increase in implementation of free-farrowing pens, we encourage further research in the direction of positive sow–piglet interactions and, subsequently, its translation into practice to enhance the potential positive implications of this behaviour for piglet and sow welfare.

## Figures and Tables

**Table 1:** Overview of data collection across farrowing groups (i.e., batch).

Methodology	Batch 1	Batch 2
Live observations	12 sows	-
Video observations	2 sows	8 sows
Data recording	Piglet-to-sow behaviour	Piglet-to-sow behaviour by litter Sow-to-piglet behaviour
	Sow-to-piglet behaviour	
	Nosing during/after suckling	
	Piglet teat order (2 to 3 time points)	
	Litter size	
	Piglet body weight, weekly	
	Piglet sex	
Data set	160 piglets	8 litters (89 piglets)
	3 time points (total 480 data points)	3 time points (total 24 data points)
	1534 piglet-to-sow occurrences	555 piglet-to-sow occurrences
	153 sow-to-piglet occurrences	83 sow-to-piglet occurrences
Analyses	by litter	by litter
	by piglet	

**Table 2:** Ethogram.

Behaviour	Description
Nose-to-nose	Piglet touches the nose disc of the sow with its nose disc
Nosing snout	Piglet touches or gently rubs the snout of the sow with its nose disc
Nosing ear	Piglet touches or gently rubs the ear of the sow with its nose disc without taking the ear into its mouth
Nosing head	Piglet touches or gently rubs the head of the sow (excluding nose disc, snout and ear) with its nose disc. Can include licking and nibbling hairs or eyelashes.
Sow-to-piglet nosing	Sow initiates contact and gently touches the piglet on any body part with her nose disc
Nosing during suckling ^1^	During nursing, the piglet stops massaging the udder, or removes itself from the udder, and touches the sow’s nose disc, snout, head or ear with its nose disc
Nosing after suckling ^1^	Within 1 min after nursing, the piglet touches the sow’s nose disc, snout, head or ear with its nose disc

^1^ Behaviour only recorded for Batch 1.

## Supplementary Materials

Data are available online at Mendeley: Camerlink, I. (2019), “Social nosing between sows and piglets in free farrowing pens”, Mendeley Data, v1 http://dx.doi.org/10.17632/3z97hv2y4x.1
